# Estimating Flexural Strength of FRP Reinforced Beam Using Artificial Neural Network and Random Forest Prediction Models

**DOI:** 10.3390/polym14112270

**Published:** 2022-06-02

**Authors:** Kaffayatullah Khan, Mudassir Iqbal, Babatunde Abiodun Salami, Muhammad Nasir Amin, Izaz Ahamd, Anas Abdulalim Alabdullah, Abdullah Mohammad Abu Arab, Fazal E. Jalal

**Affiliations:** 1 Department of Civil and Environmental Engineering, College of Engineering, King Faisal University, Al-Ahsa 31982, Saudi Arabia; mgadir@kfu.edu.sa (M.N.A.); 218038024@student.kfu.edu.sa (A.A.A.); 219041496@student.kfu.edu.sa (A.M.A.A.); 2 Shanghai Key Laboratory for Digital Maintenance of Buildings and Infrastructure, State Key Laboratory of Ocean Engineering, School of Naval Architecture, Ocean & Civil Engineering, Shanghai Jiao Tong University, Shanghai 200240, China; mudassiriqbal29@sjtu.edu.cn (M.I.); jalal2412@sjtu.edu.cn (F.E.J.); 3 Department of Civil Engineering, University of Engineering and Technology, Peshawar 25120, Pakistan; izazahmad@uetpeshawar.edu.pk; 4 Interdisciplinary Research Center for Construction and Building Materials, Research Institute, King Fahd University of Petroleum and Minerals, Dhahran 31261, Saudi Arabia; salami@kfupm.edu.sa

**Keywords:** flexural strength, beams, FRP, artificial intelligence, ANN, random forest

## Abstract

An accurate calculation of the flexural capacity of flexural members is vital for the safe and economical design of FRP reinforced structures. The existing empirical models are not accurately calculating the flexural capacity of beams and columns. This study investigated the estimation of the flexural capacity of beams using non-linear capabilities of two Artificial Intelligence (AI) models, namely Artificial neural network (ANN) and Random Forest (RF) Regression. The models were trained using optimized hyperparameters obtained from the trial-and-error method. The coefficient of correlation (R), Mean Absolute Error, and Root Mean Square Error (RMSE) were observed as 0.99, 5.67 kN-m, and 7.37 kN-m, for ANN, while 0.97, 7.63 kN-m, and 8.02 kN-m for RF regression model, respectively. Both models showed close agreement between experimental and predicted results; however, the ANN model showed superior accuracy and flexural strength performance. The parametric and sensitivity analysis of the ANN models showed that an increase in bottom reinforcement, width and depth of the beam, and increase in compressive strength increased the bending moment capacity of the beam, which shows the predictions by the model are corroborated with the literature. The sensitivity analysis showed that variation in bottom flexural reinforcement is the most influential parameter in yielding flexural capacity, followed by the overall depth and width of the beam. The change in elastic modulus and ultimate strength of FRP manifested the least importance in contributing flexural capacity.

## 1. Introduction

Concrete is the principal construction material for the building industry used in the construction of buildings and infrastructure because of its excellent construction properties considering an additional advantage of adding rebars in reinforced concrete structures. Although rebars improve the properties of concrete without modifying the cementitious properties of the matrix [1]. But susceptibility to corrosion of the rebars embedded in concrete has been recognized as one of the major durability problems associated with reinforced concrete (RC) structures. Corrosion infiltrates chloride ions into the concrete mix, which decomposes the protective layer formed on the steel surface [2,3,4]. The corrosion may not only induce an expansion pressure but may also result in cracking, crack widening, and spalling of concrete clear cover, which not only reduces the service life of RC structures but also causes a reduction in the cross-section of the rebars and weakens the load-carrying capacity of RC structures. The statistical evaluation indicates that a significant percentage of infrastructure failure globally has occurred due to corrosion of mild steel reinforcement embedded in concrete [5,6,7,8]. Also, marine infrastructure, including offshores, seaports, and subsea structures, is very important for the overall development of a country. To control the continuous deflation of natural resources and avoid transportation of natural resources to construct marine structures, sea sand and seawater may be a suitable replacement t [9]. However, its use may be hindered due to its high alkalinity and chloride content which may cause corrosion of steel reinforcement [10].

To avoid the critical problem of the high corrosion rate of mild steel, fiber-reinforced polymers (FRPs) are proposed as a potential alternative material due to their superior corrosion resistance and competent mechanical properties. Fiber-reinforced concrete also can resist corrosion and allow the direct utilization of sea sand and seawater as efficient and economical construction materials for marine structures [9,11,12,13,14,15,16,17]. FRP materials exhibit several properties, such as lightweight, and high tensile strength, making them suitable for structural reinforcement [18]. Different types of FRP, such as Carbon FRPs, Aramid FRPs, Glass FRPs, and Basalt FRPs, are available as common replacements for conventional steel reinforcement. FRPs have been subjected to experimental investigation for decades for their durability assessment. FRPs are reported to have the highest retention of tensile strength subjected to the highly alkaline solution [19,20,21,22,23,24,25]. Several factors shall be considered, including the performance advantage, materials cost, and application field, before using FRP. For instance, CFRP has better mechanical properties, and fatigue/creep/corrosion resistance. However, its high material price and low elongation at break are the main disadvantages. In contrast, GFRP and BFRP have a higher elongation at break and lower price, but their mechanical, corrosive, and creep properties are relatively poor, especially exposed to an alkaline environment [26,27,28]. Moreover, CFRP is highly recommended for higher flexure strength of RC beams, improved confinement, and lower deflection compared to GFRP and BFRP [17,29]. The performance of GFRP beams improves with the addition of steel bars. Crack width and mid-span deflection decreased by increasing the reinforcement ratio, whereas the load-carrying capacity was increased [30,31]. GFRP and CFRP showed superior effects on the load-carrying capacity, stiffness, and energy absorption of RC structural elements compared with conventional steel reinforcement [32].

The previous researchers recommended the use of a variety of AI models for solving engineering problems [33,34,35,36,37,38,39,40,41]. Modern engineering values numerical [42,43,44] and artificial intelligence (AI) models for solving complex and nonlinear problems. Earlier studies have employed AI models for estimating the flexural strength of FRP reinforced beams. Murad et al. [45] developed the GEP model and evaluated a high value of R equalling 0.977 compared to the one obtained from the ACI model (R= 0.974). It is noteworthy to mention that Murad et al. [45] based the accuracy of the model based on the value of R only. In the recent study conducted by Amin et al. [46], it was evaluated that the accuracy of the GEP model developed by Murad et al. [45] was smaller compared to the ACI models in terms of error indices. The ensemble tree-based models developed for the same problem also showed the superiority of ACI equations compared to the gradient boosting tree and decision tree models [46]. However, it was suggested that new AI models that can accurately predict flexural strength should be investigated.

Naderpour et al. [47] proposed an AI model for predicting shear resistance of FRP-reinforced concrete beams using an Artificial Neural Network (ANN). The accuracy of the proposed ANN model was more than ACI 440.1R-06, ISIS-M03-07, BISE, JSCE, and CNR DT 203. Lee et al. [48] developed an ANN model that resulted in improved statistical parameters with better accuracy than the other existing models. Random Forest (RF) was evaluated more robust model in comparison to ANN, GEP, and decision tree (DT) model [49] while investigating the compressive strength of high strength concrete. Similarly, Khan et al. [50] also evaluated the superiority of RF in comparison to the GEP model in estimating the compressive strength of geopolymer concrete. Therefore, ANN and Random Forest (RF) models, which are modern Artificial intelligence (AI) techniques, are explored in this research work to develop a simple and more accurate model that can predict the flexural capacity of concrete beams reinforced with FRP bars. The ANN model developed in this study showed higher accuracy in comparison to the previously developed AI models, therefore leading to study optimization of the ANN model in the future work.

## 2. ACI Approach towards Calculating Flexural Capacity

Numerous analytical and numerical models are available in the literature for the prediction of the flexure behavior of FRP reinforced beams [51]. ACI-440-17 [52] and the CSA S806-12 [53] have also developed guidelines for designing FRP reinforced beams. The formulation shown in Equations (1)–(5) presents the ACI model for calculating the flexural capacity of FRP reinforced beams, where, *c_b_* is the distance from extreme compression fiber to the neutral axis of the member at balanced strain condition (mm) and *c* is the distance from extreme compression fiber to the neutral axis of the member (mm). $\rho_{b}\;$ is a balanced reinforcement ratio. *f_f_* is tensile stress of FRP rebar at failure, $E_{f}$ is the elastic modulus of longitudinal FRP bars, is ultimate concrete strain = 0.003, $f_{fu}$ is the ultimate tensile strength of FRP rabars. $\rho_{f}\;$ is the FRP reinforcement ratio, $A_{f}$ is the area of longitudinal flexural reinforcement (mm^2^), *b* is the width of the beam (mm), and *d* is the depth of the beam (mm). $\beta_{1}$ is the compressive stress block parameter, and $f_{c}^{\prime }$ is the concrete compressive strength.

(1) $$M_{n}=A_{f}f_{fu}\left(d-\frac{\beta_{1}c_{b}}{2}\right)$$

(2) $$c_{b}=\left(\frac{\varepsilon_{cu}}{\varepsilon_{cu}+\varepsilon_{fut}}\right)d\;\;\mathrm{when}\;\;\rho_{f}<\rho_{b}$$

(3) $$f_{f}=\sqrt{\frac{{\left(E_{f}\varepsilon_{cu}\right)}^{2}}{4}+\frac{0.85\beta_{1}f_{c}^{\prime }}{\rho_{f}}E_{f}\varepsilon_{cu}-0.5E_{f}\varepsilon_{cu}\leq f_{fu}}\;\mathrm{when}\;\;\rho_{f}>\rho_{b}$$

(4) $$\beta_{1}=\{\begin{array}{c} 17\leq f_{c}^{\prime }\leq 28\;\;\;\;\;\;\;\;\;\;\;\;\;\;\;\;\;\;\;\;\;\;\;\;\;\;\;\;\;\;\;\;\;\;\;\beta_{1}=0.85\\ 28<f_{c}^{\prime }<55\;\;\;\;\;\beta_{1}=0.85-\frac{0.05\left(f_{c}^{\prime }-28\right)}{7}\\ f_{c}^{\prime }\geq 55\;\;\;\;\;\;\;\;\;\;\;\;\;\;\;\;\;\;\;\;\;\;\;\;\;\;\;\;\;\;\;\;\;\;\;\;\;\;\;\;\;\;\;\;\;\;\;\beta_{1}=0.65 \end{array}$$

(5) $$\rho_{f}=\frac{A_{f}}{bd}$$

## 3. Experimental Database

Table 1 lists the experiments that were used to construct the experimental database, collected from a wide range of literature, also reported by Murad, Y., A. Tarawneh, F. Arar, A. Al-Zu’bi, A. Al-Ghwairi, A. Al-Jaafreh and M. Tarawneh [45] and Amin et al. [46]. As depicted from Equation (6), flexural strength, i.e., the moment, is governed by six input factors: beam depth (*D*), beam width (*W*), concrete compression strength (*f_c_′*), area of bottom flexural reinforcement (*A_s_*), Elastic modulus of the FRP rebar (EM), and the ultimate tensile strength of rebar upon failure (*T_f_*). The data collected in terms of attributes is corroborated with the ACI formulations for flexural strength capacity [54].

(6) $$M=f\;\left(\;W,\;D,\;f_{c}^{\prime },\;A_{s},\;\;\mathrm{EM},\;T_{f}\right)$$

**Figure 1:**
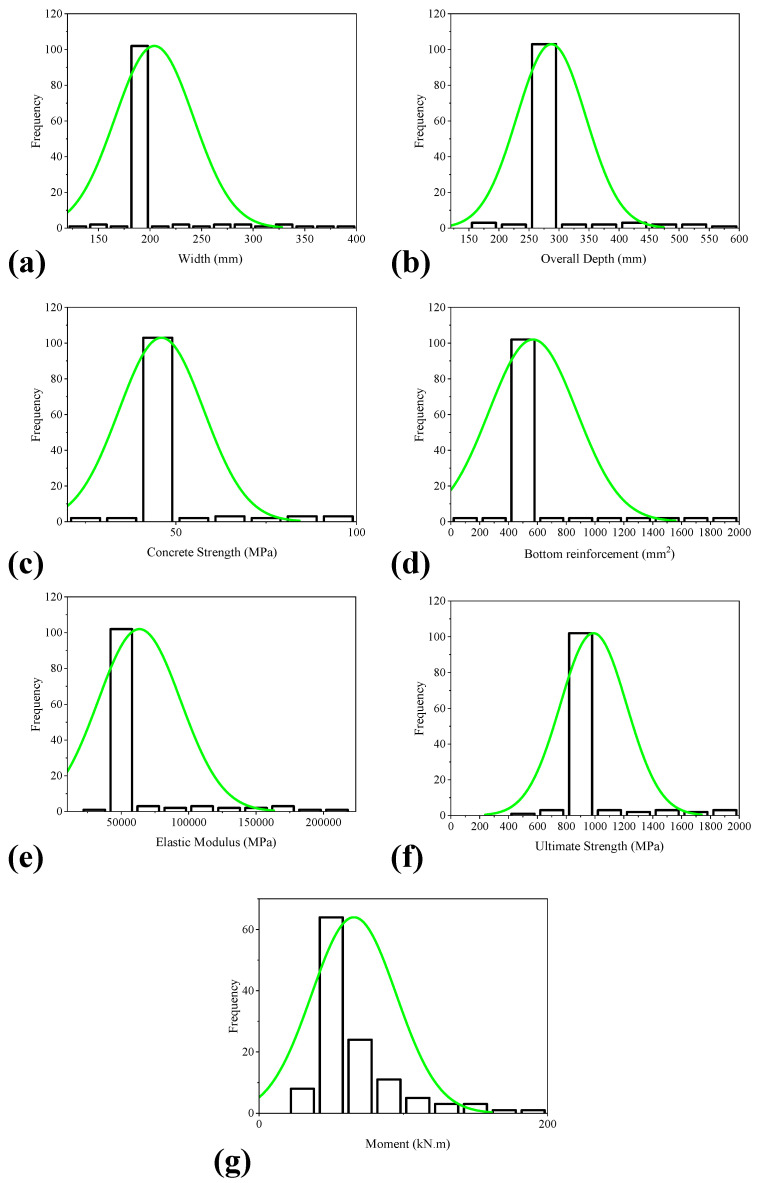
Histograms of the input and output parameters used in the current study; (**a**) Width, (**b**) Overall depth, (**c**) Concrete strength, (**d**) Bottom reinforcement, (**e**) Elastic modulus, (**f**) Ultimate strength, and (**g**) Moment.

Such that M refers to the total flexural capacity. ACI formulas demonstrate the importance of various input factors in contributing to the bending capacity. In addition, Figure 1 and Table 2 illustrate the distribution histogram and the descriptive statistics for the variables utilized here, respectively. From the histograms (Figure 1) it can be seen that the most of the samples (exceeding approximately 80%) tested in a variety of experimental works exhibit width between 130–205 mm, depth ranging from 152 to 302 mm, f_c_’ having values between 24–54 MPa, A_s_ of 57–657 mm^2^, EM of 35,630–51,260 MPa, whereas T_f_ ranges from 552 to 1152 MPa. The standard deviation values in Table 2 show that the models are constructed using a wider range of variable values.

## 4. Machine Learning Approaches

### 4.1. Artificial Neural Network (ANN)

As a bioneural network model, an artificial neural network (ANN) comes with immense sophistication to handle many complexities within any data. As an ML technique, ANN aims to mimic the knowledge accumulation and interpretation process that transpires in the human brain [73]. ANN has been extensively employed to solve nonlinear regression analysis issues [74]. The backpropagation neural network, a common ANN training approach, is often utilized in regression analysis and practical applications. In a backpropagation neural network, there are three layers: the input layer, the output layer, and a hidden (intermediate) layer linked to both input and output layers. A gradient descent algorithm, such as Widrow–Hoff arithmetic, is commonly used in backpropagation networks. Weights are modified or shifted along with the negative value of the running function’s gradient in this network. The word “backpropagation” is used to describe how nonlinear multilayer neural networks do progressive computing [75]. Despite the excellent predictive performance of the backpropagation approach, its drawback is its slow convergence speed [76]. The problem has been addressed using many optimization algorithms using simple gradient descent algorithms. Among the tested algorithms, the conjugate gradient algorithm has proven to be better than most algorithms in improving the learning time, leading to a quick convergence rate of the neural network.

The search direction for all conjugate gradient algorithms is periodically reset to the gradient’s negative [77]. The usual reset point happens when the number of repetitions equals the number of network parameters, mainly weights, and bias. In addition, to reset procedures, other methods have been used to enhance training efficiency. Concerning those approaches, it was argued by [78] relying on Beale’s version [79] that there will always be a restart in the technique process if there is a slight orthogonality change between the current and previous gradient. The following inequality demonstrates this:

(7) $$\left|g_{k-1}^{T}g_{k}\right|\geq 0.2{\left\|g_{k}\right\|}^{2}$$

where $g_{k}$ is the *k^th^* iteration’s gradient. The search direction is reset to the negative of the gradient if this condition in Equation (7) is met.

The number of neurons in the hidden layer is crucial in designing the network. Fewer neurons often lead to incomplete signal recognition or underfitting in complex datasets [80]. When the neurons are more than needed for the network, the lattice time increases, leading to overfitting because the network receives too much information or the training sub-dataset does not need enough specific information to train the network. Factors like the number of network’s input and output layers, target data noise, number of sample set cases, error function complexity, the network’s architecture, and the network’s training algorithm affect the number of hidden layers. As there is no method to quickly determine the optimal number of neurons in the hidden layers without prior training of the network [81], the trial-and-error approach is still widely adopted.

### 4.2. Random Forest Regression (RFR)

Random forest trees (RFT) employ the concept of ensemble learning, contain several decision classification trees, gather the findings by randomly picking characteristics from each decision tree, and then utilize the majority voting or averages approach to conclude. On a general note, statistical evaluation indices like correlation coefficient and error metrics (MAE, MSE, RMSE, etc.) attained by the current RF model are equivalent to those produced by other ML models like ANFIS and GEP. However, the simplicity with which RF may be used to represent categorical variables makes it a popular technique for prediction. The benefit of using a decision tree is that it can quickly model enormous datasets [82]. It can also handle both numeric and category data. According to the tree’s leaf, the base of the decision tree reflects a collection of ordered circumstances [83,84]. At the start, samples required for bootstrapping are obtained at random by replacing the existing datasets for training. RF then receives the values of various empirical characteristics, i.e., the (x) input vector. Following that, RF constructs a set of K regression trees and averages the findings. The RF equation for prediction presented in Equation (8) is arrived at after


(8) $${\hat{f_{Yf}}}^{K}\left(x\right)=\frac{1}{K}\sum_{k=1}^{k}T\left(x\right)$$

As a result of the bagging process, trees become more diverse as they grow from multiple training data subsets selected to avoid tree correlation. The Out-of-Bag (OOB) samples were not selected for the training of the k^th^ tree during the bagging process. Performance evaluation can be carried out by the k^th^ tree on these OOB elements. RF can obtain a precise generalization error estimate without using an external subset of text data [85].

The model’s capacity to generalize [75,86] can be used to assess the predictive power of the proposed model. The better the generalization ability and the lower the generalization error, the better the results from the proposed model. Numerous tree predictors are randomly selected and trained for data X and prediction set Y. hence, the mean square error (generalization) of one tree predictor *V(X)* is $E^{*}=E_{XY}{\left(Y-V\left(X\right)\right)}^{2}$ .

As the number of trees continues to increase until it gets to infinity, then the generalization error, $GE^{*}$ thus becomes,

(9) $$E_{X,Y}{\left(Y-av_{i}V\left(X,\theta_{i}\right)\right)}^{2}\to E_{X,Y}{\left(Y-E_{\theta }V\left(X,\theta \right)\right)}^{2}=GE^{*}\left(\mathrm{forest}\right)$$

where $av_{i}$ is the average value, $\theta_{i}$ is the value of a random variable, $E_{\theta }$ is the expected function, and $GE^{*}\left(\mathrm{forest}\right)$ represents the generalization error of the RF proposed model.

(10) $$GE^{*}\left(\mathrm{tree}\right)=E_{\theta }E_{X,Y}{\left(Y-H\left(X,\theta \right)\right)}^{2}$$

where $GE^{*}$ represents the average generalization error of the RF. Assuming that for any $E\left(Y\right)=E_{X}H\left(X,\theta \right)$ , then;

(11) $$GE^{*}\left(\mathrm{forest}\right)\leq \bar{y}GE^{*}\left(\mathrm{tree}\right)$$

where $\bar{y}$ is the weighted correlation between Y−H(x,θ) and Y−H(x,θ′)

## 5. Results and Discussion

### 5.1. Linear Correlation Matrix

The data used in this study were examined for linear Pearson’s correlation in order to assess the correlation between the variables. It can be observed that the flexural capacity of beams has a significant positive association with A_s_ and D equaling 0.70 and 0.85, respectively. The fundamental ACI equation for beam flexural strength shows a similar trend in the flexural capacity with rise in A_s_ and D. The width of the beam (W) and f_c_’ have a moderately positive relationship with M. Small linear correlations are depicted by the remaining properties EM and T_f_, indicating the presence of non-linear correlations between the inputs and the target variable. The detailed coefficient matrix provided can be seen in Table 3.

### 5.2. Prediction Performance of the Developed Models

**Figure 2:**
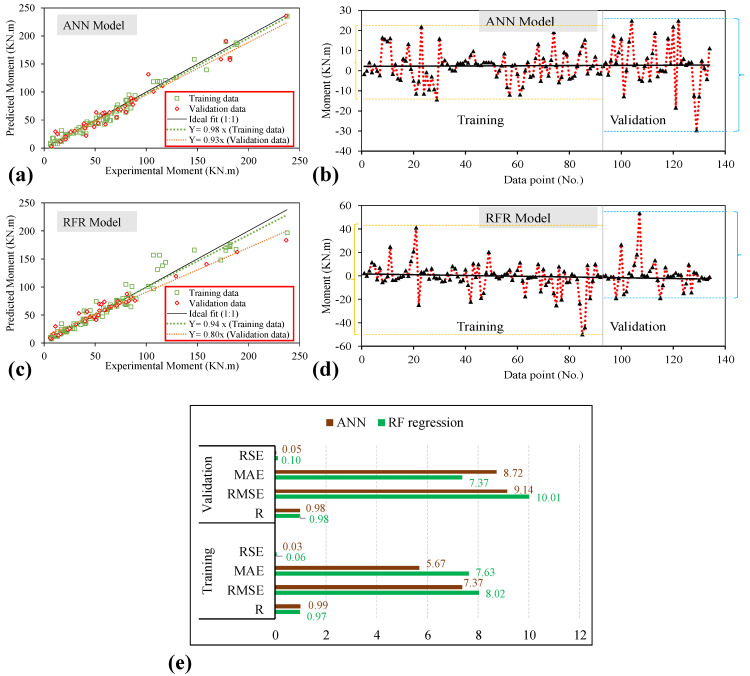
(**a,c**) Comparison of experimental and predicted results, (**b**,**d**) Error Analysis of the proposed models, and (**e**) Performance indices values for training and validation datasets, in case of ANN and RFR modeling.

The results predicted by the proposed models (ANN and RFR) are cross-plotted against the experimental data, as shown in Figure 2a,c. The estimated results from the two proposed models are strongly correlated with the experimental data, with the slope of the regression lines for the training data being 0.98 and 0.94 for ANN and RFR, respectively. As shown in Figure 2a,c, the validation datasets also reveal a strong correlation in terms of the regression line slope compared with the slope of the ideal fit (1:1). The validation dataset yields a regression line slope of 0.93 and 0.80 for the ANN and RFR models. The effectiveness and efficiency of the ML models are revealed by the closeness of their data points to the regression line (1:1). In addition, error analysis plots for the training and validation datasets Figure 2b,d have been plotted to show the range of residual errors while prediction of moment (kN.m) in contrast to the actual experimental dataset. For the ANN model, the error values range from −15 to 22 kN.m for the training data and from −32 to 23 kN.m for validation data, as depicted in Figure 2b. In contrast, for the RFR model, the error values range from −51 to 41 kN.m for the training data and −20 to 54 kN.m for validation data as shown in Figure 2d. The statistical evaluation of the generated models is illustrated in Figure 2e using four main performance indices, i.e., RSE, RMSE, MAE, and correlation coefficient. This is performed to evaluate the robustness, efficacy, as well as the relative examination of the formulated ANN and RFR models in order to predict the flexural strength of FRP, reinforced concrete beams. For robust performance and strongly linked models, the distribution of sample points has to be closer to the standard line, having a slope larger than 0.8, minimum error indices (MAE and RMSE), and R greater than 0.8 [87,88,89,90,91,92]. The values of R_training_ dataset of ANN and RFR models are 0.99 and 0.97, respectively. Similarly, the R_validation_ dataset equals 0.98 each for both the models thus suggesting that both models are strongly correlated in case of both the datasets. Since the values of both the training and validation stages are almost the same, therefore, there are no problems with overfitting in the ANN and the RFR models. It is important to note that a greater R-value is not necessarily the only tool to evaluate the AI model’s reliability [90]. As a result, we have deployed several error indices such as RSE, RMSE, and MAE in the current study. The optimization procedure primarily focuses on minimizing MAE while training the model with greater correlation metrics. The values of MAE are recorded to be 5.67 MPa and 7.63 MPa during the training stage of ANN and RFR models, respectively. The validation dataset witnessed 7.37 and 8.72 MPa for ANN and RFR models. The RSE and RMSE equals (0.06 and 8.02) and (0.03 and 7.37) in the training stage; (0.10 and 10.01) and (0.05 and 9.14) in the validation phase, respectively, for ANN and RFR models.

**Figure 3:**
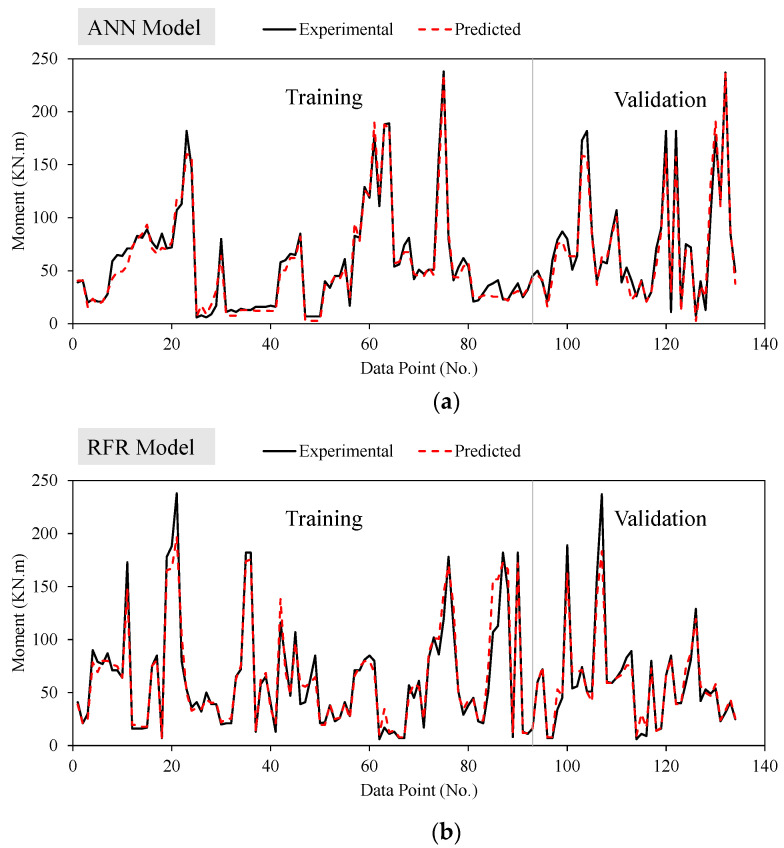
Tracing of experimental results by the prediction models (**a**) ANN and (**b**) RFR.

Figure 3 shows the tracing of experimental results by the prediction models for ANN and RFR models. It can be observed that the ANN model traces the experimental results more closely compared to the RFR model. The ANN model outperforms the RFR model owing to a higher R-value for both training and validation datasets and other statistical measures. Thus, the ANN model proves to be more robust in performance in contrast to the RFR model. As a result, in addition to having a greater correlation and lower error statistics, the proposed models may be utilized to predict the flexural strength of FRP reinforced beams, allowing designers as well as practitioners to prevent extensive testing and thus save money and effort.

### 5.3. Sensitivity and Parametric Analysis

Due to the high accuracy of the ANN model compared to the RF regression model, ANN was used to see the effect of contributing parameters in yielding flexural capacity. In order to validate the developed model, it is necessary to investigate the importance of each variable and compare its results with the existing literature. A simulated data set was therefore created, with one input variable being altered equally between its extremes while the other input variables remained fixed at their mean values (Table 4). To determine the parametric effect of a variable, the variation in the target variable was plotted versus the variable input. In the same way, the simulated dataset was used to perform the sensitivity analysis. The relative proportion of each contributing variable was calculated by normalizing the difference in the values of the target variable with regard to every input variable.

**Figure 4:**
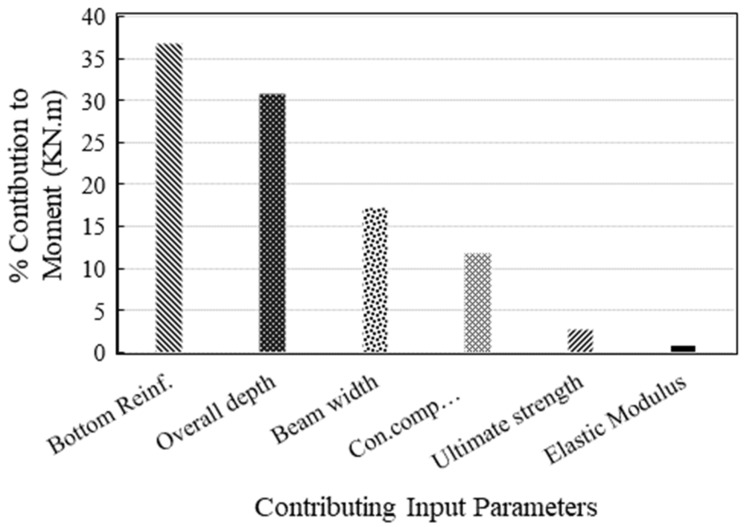
Importance of the variables reflected from the ANN model.

Figure 4 depicts the results of the sensitivity analysis. It can be observed that the results obtained in the sensitivity analysis are corroborated with the Pearson’s correlation obtained in Table 3. The variation in bottom flexural reinforcement is the most influential parameter, followed by the depth of beam among the attributes considered in this study. The width of the beam and compressive strength of concrete is the next important attributes in yielding flexural capacity of FRP reinforced beams. The ultimate tensile strength of FRP and elastic modulus are the least important parameters, which is evident from the Pearson correlation as well. Suppose we see the formulations of ACI regarding flexural capacity based on the principles of mechanics, shown in Equation (1), A_s_ is directly related to the nominal capacity. The effective depth is another important term, followed by the compression block depth, which depends on the width of the beam. Thus, the results of the developed model are corroborated with the literature; therefore, the prediction model is considered reliable for estimating the flexural capacity of beams for unseen data.

**Figure 5:**
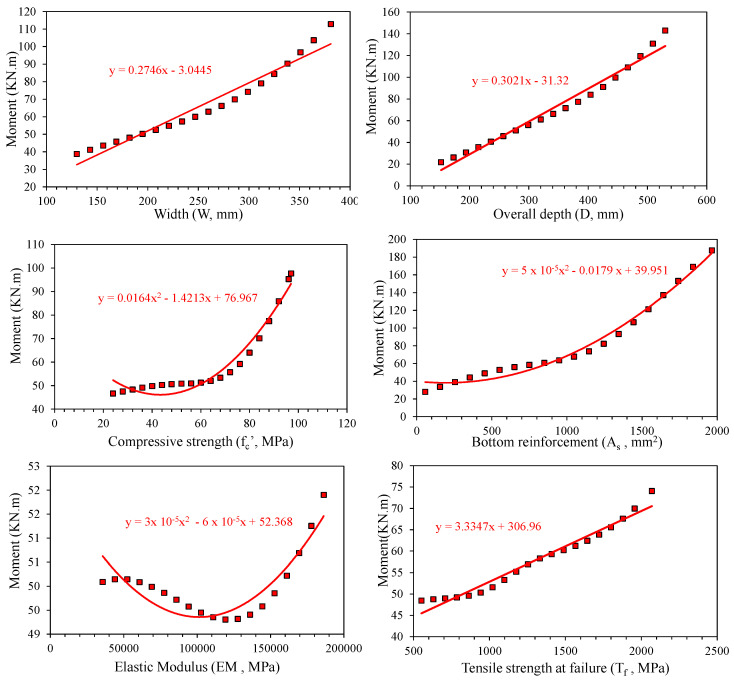
Parametric Analysis of ANN model.

Figure 5 illustrates the parametric analysis based on the ANN model. It can be noted that an increase in bottom reinforcement from 0 to 2000 mm^2^ increases the flexural capacity from 20 kN.m to 190 kN.m (170 kN.m change). The change in beam depth from 152–550 mm increased the capacity of bending from 20 to 150 kN.m (130 kN.m change). The width of the beam from 130–381 mm increased the flexural capacity by 70 kN.m. The variation in bending capacity due to elastic modulus of FRP rebar and concrete compressive strength depicted a two-degree polynomial variation. The results obtained corroborate the sensitivity and Pearson’s correlation; thus, the developed ANN model can be relied upon for future predictions. Actually, the parametric study is conducted in order to validate the trained model. The purpose was to see that the trend achieved from the trained ANN model and literature are the same. Moreover, increasing parameters like width and depth of beam, and flexural reinforcement generally increases the flexural capacity; however, optimum values depend upon many other factors like availability of clear space, aesthetics, and economy.

### 5.4. Comparison of ANN Model with ACI Formulations

The statistical results obtained previously by Amin et al. [46] and Murad et al. [45] revealed that the training data of the GEP and ACI model for the same dataset depicted a value of R, MAE, and RMSE equaling (0.977, 15.23, and 19.57), and (0.974, 4, 11.91), respectively. The Gradient Boosting Tree (GBT) model yielded R, MAE, and RMSE as 0.974, 10.7, and 17.2, respectively. It can be noticed that the GEP and GBT predicted reliably in terms of R only; however, R shall not be solely used for the evaluation of AI models. It can be noticed that error evaluation reflects the results from ACI formations as more robust compared to the GEP and GBT models. If we compare the results of the current ANN model, R (0.99), for the training set surpassed all other models; however, error evaluation depicts MAE and RMSE equal to 5.67 and 7.37, which shows that RMSE for ANN is smaller compared to the ACI formulations whereas, MAE for the ACI is smaller. The accuracy of the ANN model is more comparable to the ACI model rather than GEP, GBT, and RF regression models. This compassion suggests that hybrid ANN models optimized by a variety of Algorithms are capable of further minimizing this prediction error [38].

## 6. Conclusions

The existing ACI formulations for the flexural capacity of FRP reinforced beams, based on the principles of mechanics, are based on several assumptions that cause a difference in experimental and calculated values. Due to the availability of robust AI models, an attempt has been made in this study to estimate the flexural capacity of beams using ANN and RF regression models. Following conclusions can be drawn from this study.

- The accuracy of the existing ACI guidelines was measured using R, MAE, and RMSE with the experimental data, which yielded values equaling 0.974, 4, and 11.91, respectively. This shows that more robust models are required that can predict flexural capacity more accurately.
- The linear Pearson’s correlation obtained for the experimental data showed that bottom flexural reinforcement and depth of the beam are strong positive correlated with the bending capacity of the beam. The results from the parametric and sensitivity analysis also reflected similar interpretations of these variables. According to the ACI guidelines, bottom flexural reinforcement and depth are critical parameters in enhancing flexural capacity. Thus, the results of Pearson’s correlation, sensitivity ad parametric study, and the literature are highly matching with each other, making the developed models more reliable for future use.
- An ANN model yielded R, MAE, and RMSE of 0.99, 5.67, and 7.37, respectively whereas the RF regression model manifested 0.97, 7.63, and 8.02, respectively, for the training data. The ANN model surpassed the accuracy in comparison to the RF models as well as previously developed GEP and GBT models in the literature.
- The accuracy analysis of the ANN model is comparable to the ACI formulations, and it is expected that ANN hybrid models optimized by various algorithms may increase the performance of the ANN model. Therefore, future study is needed on the basis of hybrid ANN models to increase the prediction accuracy to a more robust level.

## Figures and Tables

**Table 1:** Summary of input and output characteristics utilized in the model formulation using ANN and RF regression.

Output Variable, i.e., Moment (kN-m)	Samples (No.)	Input Variables						
		Depth (mm)	Width (mm)	Concrete Compressive Strength (MPa)	Bottom Reinforcement (mm^2^)	Elastic Modulus (MPa)	Ultimate Strength (MPa)	References
42–81	6	305	152	29–45	355–1013	45,500–50,600	552–896	[55]
6–34	9	152–250	150–152	25–36	71–429	45,000–44,800	760–1000	[56]
81–198	9	300–550	200	43–52	573	42,000–49,000	641–689	[57]
80–182	3	300–550	43	573	600	45,000	600	[58]
39–41	4	240	200	35–36	508	43,370	885	[59]
34–57	4	210–300	200	31–41	507–1134	35,630–43,370	700–886	[60]
71–90	12	300	200	39–41	254–1013	40,000–122,000	617–1988	[61]
20–30	8	180	130	46–97	238–475	38,000	773	[62]
6–17	14	200–300	150	28–50	57–113	38,000	650	[63]
11–17	12	152–203	191–381	28	80–320	41,400	830	[64]
58–85	8	300	200	45–52	349–1046	37,600	773	[65]
49–66	6	300	180	35	253–507	40,000	695	[66]
52–54	2	300	200	24–27	88–226	200,000	1061–2000	[67]
39–85	5	270–294	200	42–54	299–1356	38,000–49,000	552–773	[68]
47–51	3	229	178	48	219–1077	41,000–124,000	552–896	[69]
14–16	2	152	152	49–52	63–99	140,000	1900	[70]
80–238	5	380	280	34–43	339–1964	38,000–40,200	582–603	[71]
81–189	12	400	200	29–73	261–1162	48,700–69,300	762–1639	[57]
49–54	3	254–256	230	40	226–603	50,000	1000	[72]

**Table 2:** Descriptive statistics of input and output variables used in the current study.

S.No.	Width (mm)	Overall Depth (mm)	Concrete Compressive Strength (MPa)	Bottom Reinforcement (mm^2^)	Elastic Modulus of FRP (MPa)	Ultimate Strength (MPa)	Moment (kN.m)
Minimum	130	152	24	57	35,630	552	21.8
Maximum	381	550	97	1964	200,000	2069	187.62
Mean	204.16	287.24	46.02	568.84	63452.00	988.78	65.75
SD	37.89	57.14	11.70	302.82	30585.99	229.96	29.22
Kurtosis	9.4853	9.3561	8.9865	9.2757	8.2132	9.5282	3.9498
Skewness	2.9951	2.7783	2.9684	3.0520	2.9973	3.0197	1.9073

**Table 3:** Correlation matrix among variables used in the development of models.

Attribute	A_s_	D	EM	fc’	T_f_	M	W
A_s_	1.00						
D	0.44	1.00					
EM	−0.17	0.01	1.00				
fc’	0.09	0.03	−0.02	1.00			
T_f_	−0.23	−0.17	0.76	0.06	1.00		
M	0.70	0.85	0.04	0.16	−0.06	1.00	
W	0.09	0.19	−0.04	−0.31	−0.04	0.22	1.00

**Table 4:** Simulated data set used for Parametric and Sensitivity Analysis.

Variable Input Parameters		No. of Datapoints	Constant Input Parameters
Parameter	Range		
Width (W, mm)	130–381	20	D = 274.40; f_c_’ = 42.85; A_s_ = 482.85; EM = 53,060, T_f_ = 927.59
Overall depth (D, mm)	152–550	20	W = 194.25; f_c_’ = 42.85; As = 482.85; EM = 53,060, T_f_ = 927.59
Conc. Compressive strength (f_c_’,Mpa)	24–97	20	D = 274.40; W = 194.25; A_s_ = 482.85; EM = 53,060, T_f_ = 927.59
Bottom Reinforcemnet (A_s_, ssqr.mm)	57–1964	20	D = 274.40; W = 194.25; f_c_’ = 42.85; EM = 53,060, T_f_ = 927.59
Elastic modulus (EM, Mpa)	35,630–200,000	20	D = 274.40; W = 194.25; f_c_’ = 42.85; A_s_ = 482.85; T_f_ = 927.59
Ultimate strength (T_f,_ Mpa)	552–2069	20	D = 274.40; W = 194.25; f_c_’ = 42.85; A_s_ = 482.85; EM = 53,060,

## Data Availability

The data used in this research has been properly cited and reported in the main text.
