# Correction: Khan et al. Estimating Flexural Strength of FRP Reinforced Beam Using Artificial Neural Network and Random Forest Prediction Models. *Polymers* 2022, *14*, 2270

**DOI:** 10.3390/polym18111349

**Published:** 2026-05-29

**Authors:** Kaffayatullah Khan, Mudassir Iqbal, Babatunde Abiodun Salami, Muhammad Nasir Amin, Izaz Ahamd, Anas Abdulalim Alabdullah, Abdullah Mohammad Abu Arab, Fazal E. Jalal

**Affiliations:** 1Department of Civil and Environmental Engineering, College of Engineering, King Faisal University, Al-Ahsa 31982, Saudi Arabia; mgadir@kfu.edu.sa (M.N.A.); 218038024@student.kfu.edu.sa (A.A.A.); 219041496@student.kfu.edu.sa (A.M.A.A.); 2Shanghai Key Laboratory for Digital Maintenance of Buildings and Infrastructure, State Key Laboratory of Ocean Engineering, School of Naval Architecture, Ocean & Civil Engineering, Shanghai Jiao Tong University, Shanghai 200240, China; mudassiriqbal29@sjtu.edu.cn (M.I.); jalal2412@sjtu.edu.cn (F.E.J.); 3Department of Civil Engineering, University of Engineering and Technology, Peshawar 25120, Pakistan; izazahmad@uetpeshawar.edu.pk; 4Interdisciplinary Research Center for Construction and Building Materials, Research Institute, King Fahd University of Petroleum and Minerals, Dhahran 31261, Saudi Arabia; salami@kfupm.edu.sa

## References

In the original publication [1], reference 31 (Vickers, N.J. Animal communication: When I’m calling you, will you answer too? *Curr. Biol.* **2017**, *27*, R713–R715) was incorrectly inserted in the References section.

Therefore, reference 31 has been replaced in the References section and reads as follows:

31.Khorasani, A.M.; Esfahani, M.R.; Sabzi, J. Effect of transverse and flexural reinforcement on deflection and cracking of GFRP bar reinforced concrete beams. *Compos. Part B Eng.* **2019**, *161*, 530–546.

The authors state that the scientific conclusions are unaffected. This correction was approved by the Academic Editor. The original publication has also been updated.
